# Determinants of adolescent pregnancy and access to reproductive and sexual health services for married and unmarried adolescents in rural Lao PDR: a qualitative study

**DOI:** 10.1186/s12884-018-1859-1

**Published:** 2018-06-08

**Authors:** Vanphanom Sychareun, Viengnakhone Vongxay, Souphaphone Houaboun, Vassana Thammavongsa, Phouthong Phummavongsa, Kongmany Chaleunvong, Jo Durham

**Affiliations:** 1 Faculty of Postgraduate Studies, University of Health Sciences, Ministry of Health, Lao PDR, Laos; 2 Faculty of Basic Sciences, University of Health Sciences, Ministry of Health, P.O. Box 7444, Vientiane, Lao PDR Laos; 3 School of Public Health, University of Queensland, Herston Road, Hesrston, Brisbane, QLD 4006 Australia

**Keywords:** Adolescent, Sexual and reproductive health, Maternal health, Determinants teen pregnancy

## Abstract

**Background:**

Early marriage and pregnancy is a risk factor for poor maternal and child health and socio-economic outcomes. Bokeo and Luang Namtha provinces in northern Lao People’s Democratic Republic (PDR) has high rates of teenage pregnancy. The purpose of this research was firstly to explore factors contributing to teenage pregnancy in rural Lao. Secondly, to understand the specific challenges adolescent mothers face in accessing maternal health services.

**Methods:**

Qualitative interviews were undertaken with adolescent mothers and unmarried adolescents aged 12 to 19 years, living in rural areas, and from different ethnic groups. In total, we undertook six focus group discussions with adolescents aged 13–19 years, twenty in-depth interviews with unmarried/married adolescents aged 12–19 years. In addition, we interviewed husbands of the adolescent mothers (*N* = 8) and mothers-in-law of both male and female adolescents (*N* = 9), community leaders and healthcare providers (health providers *N* = 17 and community leaders *N* = 12). Thematic analysis was used to analyze the data, based on a conceptual framework identified at the outset of the study.

**Results:**

The findings suggest that pre-marital sex, early marriage and pregnancy are the norm in these settings. Determinants of teenage pregnancy included liberal attitudes to teen pre-marital sexual intercourse, early marriage and pregnancy, incomplete knowledge of sexual and reproductive health and limited access to appropriate services.

**Conclusion:**

The determinants of teenage pregnancy in this setting are multi-dimensional, and require a range of responses. As some of the determinants are deeply embedded in the system of local values, beliefs and practices, and form part of the logic of what it is to become a healthy woman, these practices are deeply entrenched and may be resistant to new knowledge. The challenge therefore is to find culturally responsive strategies that enable individual and collectively agency.

## Background

Globally, adolescent pregnancy is the second leading cause of mortality in the 15- to 19-year-old age group and is major public health concern [1, 2]. An estimated 16 million girls aged between 15 and 19 give birth every year with over 90% of adolescent pregnancies estimated to occur in low- and middle-income countries, making up 11% of all births globally [2, 3]. While data is often incomplete, available data suggest regional differences with births to adolescents as a percentage of all births ranging from an estimated 2% in China to 18% in Latin America and the Caribbean [4]. High rates of adolescent pregnancy are of concern, as early sexual debut and pregnancy poses increased risk of poor maternal and infant outcomes across a range of indicators [5–9].

Adolescent maternal mortality is estimated to be about a third higher among adolescents than among 20- to 24-year olds [10]. Adolescent maternal mortality and morbidity is due to complications during pregnancy and childbirth, include higher rates of hypertensive disorders of pregnancy, anemia, gestational diabetes, co-morbidities and complications during delivery compared to adult women [11, 12]. Adolescent pregnancy is also associated with higher rates of low birth weight, preterm delivery, respiratory diseases and infant mortality [11]. Pregnancy during adolescence can also contribute to poorer financial, emotional, social and health outcomes, often compounded by generally sub-optimal nutritional intake and limited access to antenatal and postnatal care [1, 11, 12]. Adolescent mothers also tend to leave school early, limiting future employment opportunities [13, 14]. Early pregnancy and motherhood is also linked in some societies to negative stereotypes and stigma, which can further contribute to poor health and social inequities [15]. In other societies however, it is important to note that marriage and motherhood in adolescence is often normative and valued and important in understanding adolescent’s sexual behaviors [16, 17].

The Lao People’s Democratic Republic (PDR) is a lower-middle income country situated in South East Asia. The country is in the process of rapid economic growth and demographic and epidemiological change. It has a youthful population with approximately 60% of its over 6 million inhabitants estimated to be less than 25 years of age. The country made good progress against many of the Millennium Development Goal (MDGs) targets, including improvements in education and maternal and child health, but improvements have been uneven and deep inequities exist [18]. The maternal mortality ratio (MMR) has been reduced by 75% to 197 (80% CI = 136–307), but remains one of the highest in the region and there is considerable uncertainty regarding the MMR estimates [18]. Entrenched differentials in maternal health outcomes are evident, with ethnicity, wealth quintile, geography and education levels key determinants of poor maternal outcomes and social exclusion.

Childbearing often begins early in Lao PDR and, at 94/1000, the country has one of the highest adolescent pregnancy rates in the region [19]. This early start to child rearing is particularly evident in rural areas, where the adolescent fertility rate is estimated at 114/1000 girls aged 15–19 years [19]. Early childbearing and higher fertility rates are also correlated with lower education levels, lower wealth quintiles and ethno-linguistic group [19]. Two of the Northern provinces have been found to have a particularly high adolescent birth rate. The adolescent birth rate in Bokeo, for example, is 149/1000 adolescents, and in Luang Namtha province it is 124/1000 adolescents [19]. These two mountainous provinces are primarily rural and are characterized by great ethnic diversity, with limited access to appropriate and affordable healthcare.

These high rates of adolescent pregnancy are of concern given the increased risk of poor maternal and infant outcomes across a range of indicators [5–9]. In rural areas, age-related pregnancy risks are often compounded by malnutrition, poor socioeconomic conditions and low levels of literacy and limited access to maternal healthcare. Despite this, few studies in Lao PDR have focused specifically on adolescent mothers’ access to sexual and reproductive health (SRH) and maternal health services. Even fewer studies have focused on the specific needs of different ethno-linguistic groups in rural areas. This is an important gap because the important socio-cultural differences in marriage and childbearing practices make extrapolating evidence from other settings, including from the dominant Lao-Tai ethnic group, challenging. It is particularly pressing now as rural areas in Lao PDR are being radically changed by rapid modernization processes with the potential for minority peoples’ sexuality to be exploited and integrated into a market economy.

Within many of the ethnic minority groups, while marriage is usually a necessity before a childbirth, pre-marital sex often with multiple partners is considered the norm. After marriage, women are expected to have sexual relations only with their husbands, although if divorced, they may take multiple partners. Married men on the other hand, may engage in extramarital sexual relations with unmarried women, who especially if they become pregnant, may become second wives. While pre-marital sex has previously been mainly with people from within the village or surrounding village, increased integration into the market economy combined with the perception of minority girls’ promiscuity, is contributing to men from outside these ethnic communities seeking opportunities to engage in sexual liaisons with local women, often in exchange for money or other material goods [20]. The increasing demand for commercial sex and the changing aspirations of young ethnic girls is also contributing these girls engaging in transactional sex as a step to more material lifestyles [20]. The expansion of these adolescents’ sexual networks beyond ethnic and national boundaries further underscores the need to understand their particular sexual and reproductive healthcare needs. The purpose of the present study, therefore, was to better understand adolescent mothers’ health-seeking practices and their determinants in two of the most remote and most ethnically diverse provinces in the Lao PDR, namely Bokeo and Luang Namtha. The intent was to provide policy makers with information to help design appropriate interventions/programs for married and unmarried adolescent mothers, in order to inform policy and practice development through the provision of locally-relevant evidence.

## Methods

### Design

Following a detailed review of the available data describing adolescents’ access to SRH interventions and outcomes in the Lao PDR, qualitative methods were used to investigate adolescents’, community leaders, mothers-in law and service-providers’ perceptions and barriers and enablers related to adolescent access to maternal health and SRH services. The two largely rural Lao provinces of Bokeo and Luang Namtha in northern Lao PDR were selected for the present research due to their high rates of adolescent pregnancy and their ethnic diversity. As discussed under “study setting”, for this study, out of the five districts in Bokeo province, Meung district was selected while Long district was selected out of the five districts in Luang Namtha province. Both of these districts were selected based on their high rates of teenage pregnancy, high rates of poverty, remoteness and their multi-ethnic makeup [21].

### Study setting

Long district is home to more than 11 ethnic groups, and 5644 families, of which 1250 are classified as living in poverty, with 43 of the 78 villages considered poor. Thirty per cent of the villages are located in mountainous areas with limited all-year road access [22].

Bokeo province is the smallest province in the country and forms part of the “Golden Triangle” (at the crossing with Myanmar and Thailand). There are eight ethnic groups living in Meung district. The district has a total population of 14,239 with 3504 families, of which 933 are classified as living in poverty [23].

### Study participants and sampling

The study population was adolescent mothers, pregnant adolescents and unmarried adolescents aged 10 to 19 years, living in rural areas and from different non-Lao-Tai ethnic groups. We specifically focused on non-Lao-Tai ethnic groups due to their particular vulnerabilities, high rates of adolescent pregnancy and inequalities in access to healthcare. Purposive sampling was used with the specific inclusion criteria for the individual in-depth interviews (IDIs) and focus group discussions (FGDs) being: 1) Adolescent mothers or who were pregnant and 2) Adolescent girls (not married or in union), with participants having to fulfill at least one of the criteria to be eligible to participate in the study. Key informants were husbands of the adolescent mothers and their mothers-in-law (of both adolescent husbands and wives), village heads, Lao Youth Union (LYU), Lao Women’s Union (LWU), elderly, directors of district and provincial health offices and hospitals, and health center staff and village health volunteers/Traditional Birth Attendants (VHVs/TBAs). All invited participants agreed to participate in the study. We specifically selected mother-in laws because of the role they play in determining access to maternal healthcare, especially as some included communities are patriarchal. In Akha society for example, a wife is incorporated into her husband’s lineage after marriage and has no claims on children in the case of divorce because the children belong to the husband and are under protection of his ancestors. Similarly, for most of the other ethnic groups, following marriage, a women will move to live with her husband [20]. The Kui however have a matrilineal system, and the husband usually moves to live with his wife’s family at least in the early years of their marriage.

Potential participants were identified in discussion with health center staff, VHVs, Village Heads and LWU, who were first briefed on the objectives of the study and the eligibility criteria for recruitment into the study. Working with these key people, we also used the household register books to identify potential participants.

### Data collection

Face-to-face FGDs and interviews were undertaken by trained qualitative researchers from the University of Health Sciences, Lao PDR. All researchers have experience and an interest in working in sexual and reproductive health and working with adolescents. Prior to commencing the FGDs and interviews, all participants were informed of the objectives of the study. All FGDs and interviews were audio recorded with informed consent and followed an interview guide based on the study objectives. In total, six FGDs, with three in each district, were conducted. Per district, one FGD with unmarried adolescents was conducted to talk about teenage pregnancy, early marriage, education and access to family planning. In addition, per district, one FGD with adolescents who were mothers or were pregnant at the time of the study was conducted. These FGDs were facilitated by two trained female researchers. In each district one FGD with adolescent boys or husbands, facilitated by two trained male researchers, was conducted. In addition to the FGDs, 37 IDIs were conducted; twenty of these were with unmarried and married adolescents, and nine were with adolescent mothers and were conducted by the female researchers. Unmarried and married adolescents were asked about their access to and perceptions of SRH and maternal health services. Eight IDIs were conducted by the male researchers, with adolescent husbands and nine interviews with mothers-in-law, were conducted by the female researchers. These interviews aimed to understand the husband’s and mothers-in-law’s perspectives of female access to services and adolescent pregnancy. For the purpose of this study, SRH included sexually transmitted infections (STIs), including human immunodeficiency virus (HIV) and family planning, while maternal health included ANC and PNC and birthing. The FGDs and IDIs allowed us to capture a wide range of views and different experiences, providing insight into attitudes, perceptions and access to care.

In addition to the interviews with adolescents, 27 key informants were interviewed from the provincial health department (*n* = 4), district health department working in the maternal health sectors (*n* = 3), health center staff (n = 4), village leaders (n = 4), village health volunteers or TBAs (n = 4), LYU (n = 4), LWU (n = 4). In total, 15 healthcare providers and 12 community leaders were interviewed (Table 1). Key informants were asked for their perspectives related to adolescent pregnancy and access, use and barriers to SRH and maternal health services among adolescent mothers. Each FGD was made up of 6–10 participants and normally lasted for about two hours, while the individual interviews lasted approximately one hour. All interviews and FGDs were held in venues that were convenient for participants and ensured confidentiality. The sample size allowed us the reach data saturation that is when no new information was forthcoming. Field notes were taken during the research with the researchers comparing notes at the end of each day.

**Table 1:** Summary of research instruments (FGDs and IDIs)

District	Long	Meung	Total
FGDs	3	3	6
Adolescent girls (not married or in-union)	1	1	2
Adolescent mothers or who are pregnant	1	1	2
Adolescent boys and husbands	1	1	2
IDIs	34	32	66
Pregnant adolescents	10	10	20
Husbands	4	4	8
Mother-in-law	5	4	9
Healthcare providers (2 provincial, 2 districts, 4 HC, 4VHVs/TBAs)	8	7	15
Community leaders and LYU/LWU	6	6	12

For the key informants who did not speak Lao language, local translators were used to translate the verbal dialect language to the Lao language. All field notes were transcribed into Lao and then into English. For this reason, quotes are edited rather than verbatim quotes.

### Data analysis

Thematic analysis was used to analyze the qualitative data based on a conceptual framework of access to healthcare identified at the outset of the study [24]. All field notes were transcribed into Lao and then translated into English. The principal investigators (VS and JD) read all the transcriptions several times to get an understanding of the patterns of text. The texts were then coded, and compiled into categories and themes by the two principal investigators [24, 25]. The key predetermined themes were based on those identified in the literature, such as socio-economic, financial, cultural and gender roles, decision making power, knowledge on maternal healthcare, accessibility, quality of care, negative attitudes of providers, peer and family support; however, this did not excluded the possibility of new themes emerging [24, 25]. Throughout the analytic process, the entire data set and the coded extracts were crossed checked and verified.

### Ethics

The National Ethics Committee for Health Research, Ministry of Health, Lao PDR approved the research protocol. Given low levels of literacy in Lao language in these communities, verbal consent was obtained from each participant prior to interviews with the approval of the National Ethics Committee for Health Research. Typically, participants aged under 18 years need parental consent; however, in this context, most of the participants were already married and having children, so their individual consent was appropriate. Personal consent for those who were not yet married was also considered appropriate and was approved by the National Ethics Committee for Health Research, where the person had sufficient understanding to provide consent, had been counselled and did not wish to involve their parents in the consenting process. All participants were assured of privacy and confidentiality, the potential risks and benefits of the research and that they were free to withdraw from the research at any time without penalty. They also were informed and consented to the findings of the research, including extracts from the interviews, being used in any reports or publications emanating from the research.

## Results

### Determinants of early sexual debut, marriage and of teenage pregnancy

Many of the participants felt that the number of teenage pregnancies was declining, partly as a result of better knowledge of, and access to, contraception and better understanding of the risks, as well as girls wanting to stay in school longer and complete their education. Nevertheless, while no specific data were available on the prevalence of early marriage and pregnancy, almost all the participants considered initiation of sexual intercourse by fifteen as the norm, part of everyday practice and an important determinant of teen pregnancy, with parents typically holding favorable attitudes towards teen pregnancy. In these rural areas, many adolescents are either farming or working as laborers by 14 and married with children by 18, as one male adolescent husband explained:

*“It is our culture that when we grow up, we work and then we love and get married. We don’t go to school; we work for money farming or as labors since we are 14 years old. When we are thinking that we are ready, we can get married.” (Husband).*

Other determinants of early initiation of sexual intercourse, early marriage and childbearing were often attributed by participants to the traditional customs and liberal attitudes that many of the different groups held towards pre-marital sex. This was reported to be particularly the case for those of the Sino-Tibetan ethno-linguistic group and, in particular, those from Akha and Kui communities. In these communities, traditional practices allow for relatively free pre-marital sexual relations and multiple sexual partners before marriage, with adolescent boys and girls being told by family and other community members including elders, that engaging in sexual intercourse is an important part of growing up. The traditional Akha practice of *‘Breakthrough Vagina’,* which is typically the first pre-pubertal sex act for young female adolescents and involves an older, sexually experienced man having penetrative vaginal sexual intercourse with the girl, and is an important stepping-stone to adulthood and maturation, also continues and, as one health worker observed, can cause injury to young adolescents:

*“Sexual custom by Breakthrough Vagina of the girls aged 12-14 years, thus having sex at this young age lead to the tearing of vagina. There are about four cases of torn vagina each year.” (HCP).*

Early motherhood was also seen as a way of helping adolescent girls mature into adults and also acted as a determinant of early marriage pregnancy, with unmarried adolescents seen as being ‘carefree’ with limited responsibilities. A number of participants felt, for example, that there was economic value in early marriage, as it brought an additional labor unit to the household and the subsequent children would become economic assets able to help look after the livestock and work in the fields. One woman felt that because of poverty, adolescents had limited choices except to drop out of school and get married, as she explained:

*“. . some families can’t offer their children the opportunity to continue the study, especially the adolescent girls have to drop from school to get married in order to assist the family.” (Mother in Law).*

While reported as being common, few participants felt there were health advantages to early pregnancy for either the mother or the newborn. Generally, unmarried adolescent girls were seen as being healthier and stronger than those who were married. Potential health risks that were identified included a high risk of maternal and child mortality, premature delivery, post-partum hemorrhage and, for unmarried adolescents, the risk of unsafe abortion, as well as young mothers.

*“. . not knowing how to feed and rear their children properly.” (Unmarried adolescent).*

One of the young husbands noted:

*“I don’t know any advantages of early childbearing - the young mother is not healthy and the baby is also weak, because the body of girl-mother is not as ready as adult.” (Husband).*

For some, early marriage and pregnancy were seen having potential negative impacts on future socioeconomic opportunities for the adolescents and their families, especially as it meant dropping out of school.

### Unintended pregnancy, abortion and STIs

Unintended adolescent pregnancy, usually due to contraceptive non-use, inaccessibility or avoidance of birth control practices, was recognized by many of the participants as a concern. The extent of unintended adolescent pregnancy, however, was difficult to gauge as, despite the generally liberal attitudes towards pre-marital sex, pregnancy out of marriage was considered taboo. According to participants, it would be impossible for unmarried adolescents to give birth in, or remain in, the community with their child as an unmarried mother. With abortion also legally restricted and generally prohibited within the community, unmarried pregnant adolescents were expected to marry the boy who caused the pregnancy before the child was born, particularly in Akha communities. For the Akha, where the father cannot (for example is already married and cannot take a second wife) or refuses do this, he must make a sacrifice and then the responsibility of finding the girl a husband falls to the parents. In such cases, the parents will try find an Akha male in the village who is willing to marry the girl. Alternatively, she may become a second or minor wife to a married man, or a partner across the border in China, where the language and customs are similar, may be found for her. For unmarried adolescent mothers of the Kui ethnic group, if they do not marry, they have to “give” their baby to relatives, although in reality they, the mothers, still raise the child. The women themselves can subsequently marry if she chooses.

Given the above, not surprisingly, most of the adolescents insisted they had never seen an unmarried adolescent mother in their community, and most felt it would be almost impossible for a pregnant, unmarried adolescent to present at a health facility. Not surprisingly, given the illegality and taboos surrounding abortion, it was considered rare, but not unheard of. Many of the adolescents, however, were very unclear on where to go for an abortion, how to access such services, and whether the public health facilities provided abortion services or not. A few, however, were aware of the possibility of medical abortion using pills available in the market through China. According to one healthcare worker, who had seen some of the complications of illegal abortion:

*“Some of them get pregnant and have an abortion somewhere outside, and come to hospital with complication. They normally use abortion pills, and suction at an illegal clinic.” (Health Care Provider).*

In general, the unmarried female adolescents from both districts felt that their boyfriend or partner should make decisions about contraceptive use. Many were also concerned that if they took the lead in decision-making they could risk losing their boyfriend.

*“I think women do not have power on deciding to use contraception because men know better than us. Because I was afraid that my boyfriend will leave me, and he will not propose to me so I have to depend on him.” (Unmarried adolescent).*

This lack of empowerment, however, was not felt by all of the adolescents, and one unmarried adolescent explained:

*“I think [I have] a major role in [decision-making] because nowadays women have the right to decide to have a baby and when should we have a baby and also the spacing between each child.”(Unmarried adolescent,)*

While no data were available, the prevalence of STIs was often mentioned, including among sex workers, further suggesting poor accessibility or avoidance of condoms. One healthcare worker said that she saw *“about 6-7 adolescents aged 14-15 years per month” (HCP)* with genital warts. Despite often having multiple sexual relationships before marriage, once married, females are expected to be monogamous; the same is not true for men, however, and healthcare workers reported treating both married and unmarried adolescent females for STIs. One husband from Long district mentioned HIV/AIDS as the main SRH problem in his community, but this perception may be because he was aware of one HIV case in his community.

### Demand-side and supply-side barriers in adolescent access to sexual, reproductive and maternal health services

According to the adolescent mothers in our study, low utilization of sexual and maternal health services was common, and adolescent specific services were unavailable. This was also corroborated by the healthcare workers, who reported low uptake off sexual and reproductive health services, especially among unmarried adolescents. Most adolescents were reported to give birth at home, despite their increased risk of adverse outcomes. The main demand- and supply-side factors of barriers in accessing sexual, reproductive and maternal health services among adolescents and adolescent mothers related to socio-cultural norms, healthcare worker attitudes and skills, real or perceived lack of confidentiality, cost, decision making capacity and knowledge.

### Socio-cultural norms

Despite the general community acceptance of, and even encouragement of, pre-marital adolescent sex, socio-cultural norms prevented discussion of sex and reproduction. This contributed to young people’s limited understanding of sexual health, as well as reluctance to discuss SRH with providers, concern about being asked sensitive questions and of physical examination, especially by a male doctor. These factors were compounded by either limited Lao language or a lack of confidence in using Lao. For unmarried adolescents, these barriers were particularly acute, and they worried about if they would be asked about their boyfriend or why they were not married. Going alone to the healthcare facility was also considered “impossible” and a barrier for unmarried adolescents particularly. While this was less of a barrier for married adolescents, their access was constrained by the ability of their husband or another family members, being able to accompany them. Some of the reasons for needing to be accompanied related to social practices, but also to the logistics of reaching and navigating healthcare services. The girls’ husbands, for example, were often able to drive the girls to the services on their motorbike, as well as provide protection and help translate, as typically the healthcare workers only spoke Lao language. In some cases, due to work demands, husbands were not available to accompany their wives, and in-laws were reported to not always be supportive or be able themselves to accompany the adolescent, which meant that married adolescents often missed their ANC or PNC appointments. While other people could accompany the adolescent, including other family members such as older sisters and aunts, it was often harder for female members to do so, due to language difficulties and limited access to transport.

Almost all of the married adolescent mothers or pregnant adolescents said they had used the ANC services at least once, and usually the ones nearest to their homes, but none had received the government recommended four visits. As the following excerpt helps to highlight:

*“I received ANC once when I was pregnant at 4 months. I went to the district hospital with my husband’s sister. I am now at 6 months but I don’t know if I should go again or not and I don’t know when I should go for ANC again. The doctor did ultrasound and gave me some supplementary drugs and they have already run out for more than a month. I don’t know what to do. I told my husband and parents in law, but they did not do anything; I don’t know why. But my parents in law were the first ones to tell me to go for ANC because I told my husband that my menstruation had stopped for months”* ***…*** *(Adolescent).*

### Healthcare worker attitudes

Many of the participants described the unfriendliness of the healthcare staff and were particularly worried they would be scolded’, especially if they were unmarried. Providers themselves acknowledged that they were often uncertain of how to deal with adolescents, especially those from the ethnic minority groups, and said they sometimes found them very frustrating to deal with.

*“I do not feel comfortable in working with adolescent girls on their SRH needs. When adolescent girls came to use health services, I had to spend more times to communicate with adolescent girls because I did not understand their language and they are still young like children, so they do not understand well, especially if they do not have any experience in the prevention of pregnancy.” (HCP).*

All of the healthcare staff interviewed, however, indicated they had not received any training specific to working with adolescents, and they all indicated willingness to attend the training on adolescent–friendly services. Some adolescents and key informants felt that the providers lacked skills, as well as the facilities having a general lack of equipment. This was of particular concern for pregnant adolescents, as it meant in the case of a complication they may have to go to the provincial hospital which was far away and costly, both in terms of fees and travel costs. Even where services were nominally provided free, informal payments and costs for medication were often unknown in advance and could prevent people from presenting.

### Lack of confidentiality and privacy

Many participants described unmarried adolescents’ anxiety of others in the community finding out they had attended SRH services. In particular, they were afraid of losing their reputation and being gossiped about. The lack of privacy at hospitals and local healthcare facilities was frequently raised as an issue of concern, resulting in fear of being seen by friends, relatives or community members.

Typically, unmarried adolescents said that they would first self-medicate by going to the pharmacy in the case of suspected STIs, only visiting the health facility if the problem did not resolve itself. Similarly, unmarried adolescents preferred to buy their contraceptives from the pharmacy. Typically, however, unmarried female adolescents relied on their partner to purchase and make decisions about contraceptive use as the following interview excerpts help to illustrate:

*“I have brought condoms at the small grocery along the street and drugstore in Long district because my partner or boyfriend called me and said that he will come to visit me and asked me to prepare the condoms as he had no money. When I bought condoms, I felt shy. I went to the drug store and asked for condoms, the seller gave me and I was in hurry up to walk out from the drugstore because I was afraid to be seen by others and I felt shy. However, I have to use condoms. If I did not have condoms, my boyfriend will not come to see me, so, I decided to buy condoms. For the small grocery, I just did like I wanted to buy some soap and shampoo, then, I also got condoms and I paid and I did not look at the face of seller because I felt shy. I did not buy at my village and I went to buy in other villages.” (Unmarried Adolescent).*

*“It is most convenient to get condom in drug store. It is not hard to ask for because it is a place to sell and buy. If you go to health center, you will be asked many detailed questions such as why you want to get it.. . .. If you are an unmarried adolescent, the providers are more likely to speak not nicely and get angry and they look at adolescents as a stranger, so we do not dare to go to use health services at the health facilities as we are shy and we are afraid that the health provider will scold us.” (Unmarried Adolescent).*

### Cost and availability of services

There was general agreement that the costs of services, medicines and transport, as well as opportunity costs, were barriers for many adolescents.

*“….. The district hospital is more comfortable because this is near our community. The HC is far from our community about 20 km. If the provider are not skillful in technical areas, so they prefer to go to the provincial hospital which is far and we did not have transportation and budget to go.” (Married adolescents).*

*“Most of people are worried about financial problems because they are afraid they won’t have money and the doctor will leave them die alone. Thus they decided not to use the health services.” (HCP).*

### Decision-making power

Unlike many of the unmarried adolescent girls, who mainly relied on their partner to make decisions about their sexual and reproductive healthcare needs, most of the married adolescents explained that accessing services and making decisions about contraception was something they discussed and decided together with their husbands. They also recognized that parents and parents-in-law could be partners in the decision-making process because of their experience.

*“She did not have power to decide in selection of place to delivery because husbands and parents had more power to decide as they know about place of delivery better than her.” (Mother in law).*

For the decision on whether a pregnant unmarried girl would be able to go full term and where she could deliver the husbands and mothers-in-law felt this would depend on her parents and elders in the community:

*“She could not decide the place of delivery that elders in the village will select the place of delivery as delivery at HC or district hospital and not allow to deliver at home.” (Mother in Law,)*

*“I think her parents decide, because they must care for her safety.” (Husband,)*

### Lack of knowledge and practical experience

The adolescents in this study demonstrated some knowledge of SRH and maternal health and were aware of contraceptive methods (mainly condoms and the oral contraceptive pill). While they had some ideas about the types of SRH and maternal health services provided in the health services, they were often unsure about the exact nature of the services and, due to a general lack of interaction with the services, were concerned about what would happen at the clinic and what kind of questions they might be asked. Furthermore, when they did attend services, they were often too shy to ask questions when they did not understand or worried that they would be scolded, resulting in them sometimes not understanding what medication they had been given, for what purpose, or when and how to take it. Shyness, lack of confidence and limited understanding of the potential benefits of birthing in a facility, as well as the routine, everyday practices of birthing at home with one’s family, with the hospital a place of last resort, also prevented people from seeing a need to birth in a facility, as one of the mothers-in-law explained:

***“*** *Females are shy and scared to seek care or get advice with health care provider, they don’t want to open their vagina so they give birth at home with assistant of their mothers and relatives without feeling shy as they already know each other. Many people gave birth at home, and nothing happens and the new born can get healthy as children who were born at hospital. Most old people (parent) ask their daughters to give birth at home first and they will come to hospital only when there is problem.” (Mother in law).*

*“I decided to deliver at home because I felt confident that I could deliver at home because my first child, I also delivered at home with the assistance of my parent in law. There were no any problems of delivery at home with the assistance of my parent in law in our community.” (Married Adolescent).*

None of the married adolescents had attended PNC, and very few knew about it or felt there was a need. Typically, the mothers-in-law had very little understanding of the need for PNC. While the husbands seemed to have more awareness of PNC, they also felt there was no need to attend PNC if there were no obvious problems.

## Discussion

Addressing adolescent SRH is essential in reducing maternal and new born mortality and morbidity, as well as the often negative socio-economic consequences of early sexual debut and childbearing [8, 26–29]. This study has identified key determinants of early marriage and childbearing, as well as demand- and supply-side barriers facing adolescents in northern Lao PDR, with a particular focus on adolescents from different ethno-linguistic groups to the majority Lao-Tai group. This is important because these adolescents experience significant disparities in health and socio-economic outcomes. Without interventions informed by the needs and perceptions of these adolescents, these disparities are likely to continue to grow at a time when the Lao PDR is going through rapid socio-economic change, greater interaction with markets, and changing aspirations [20, 30, 31].

While the broader socio-economic context is rapidly changing, the distinctive cultural practices and understanding of what it is to be an adolescent are changing more slowly and are deeply embedded in the social norms of previously remote communities, such as those included in this study. In this context, adolescent marriage and pregnancy is the norm, with marriage after the age of twenty considered undesirable and indeed, many were concerned that if they were not married by twenty they would not be attractive to men. From this perspective, early initiation of sexual intercourse, marriage and childbearing need to be understood within the cultural logic of the sexual practice of what it is to be an adolescent in these communities and the taken for granted reality [32].

There was substantial agreement between the accounts of the different participant groups included in this study, with participants identifying a range of supply- and demand-side barriers to adolescents accessing sexual, reproductive and maternal health services. Many of these related to the well documented supply- and demand-side barriers of affordability, acceptability, availability and accessibility that interact in complex ways with power imbalances, perceived need and the capacity of the girls to make, or act on, their own decisions about service use, as well as language barriers. In addition to these barriers, unmarried female adolescents, however, experienced specific barriers related to their young age and, more specifically, their marital status. Shyness, lack of experience and confidence in interacting with healthcare facilities, staffed primarily by people of Lao-Tai ethnicity, reputational concerns, limited autonomous decision-making capacity, and knowledge gaps were particularly acute for unmarried adolescents and limited their capacity to control their reproduction, protect themselves from STIs and utilize healthcare services. As has been observed elsewhere, [33–38], the real or perceived lack of confidentiality and judgmental attitudes of service providers were also strong disincentives for unmarried female adolescents to seek care.

The skills and attitudes of providers were also identified as barriers to access for both married and unmarried adolescents. The healthcare providers in this study also recognised the need for them to be provided specific training in relation to the SRH needs of adolescents. Inadequate training has been associated with negative attitudes towards adolescent SRH [37, 39]. Training that addresses knowledge, attitudes and communication and counselling skills (including confidentiality) may enhance provider skills, and assist in shifting attitudes to those that are more understanding of adolescents [39, 40]. This is important as forming trusting relationships between adolescents and healthcare practitioners is likely to be essential in contributing to sexually healthy development of adolescents as they transition to adulthood.

Few of the adolescent mothers reported attending the recommended number of ANC or PNC visits or delivering in a healthcare facility. This was mainly due to the aforementioned demand- and supply-side barriers and, in particular, perceived need, lack of knowledge about the potential benefits, and the high cost. Even where facility based birthing is free of charge, there may still be unpredictable informal charges, as well as the costs of transport and potential loss of income for at least one person who needs to accompany the adolescent female to the facility. A similar finding was observed in ethnic communities in Vietnam [41].

Community support is an important predictor of adolescents’ health-seeking practices and, given the importance of socio-cultural norms in influencing sexual and healthcare seeking practices, engaging with communities, particularly gate-keepers such as parents and community leaders, is likely to be key to any effective intervention. Also important is increasing adolescents’ and their families’ knowledge and demand for SRH and maternal health services, and which includes the participation of communities and adolescents in the design and implementation of interventions as the norm, recognises the underlying cultural philosophies, and draws on the strengths and capabilities already present in the different communities [42]. The study suggests the need for greater emphasis in health promotion activities which empower individuals to be active agents of change. This needs to include developing both knowledge and communication skills in adolescents so that they can take more control in contraceptive decision-making as well as deliberately creating opportunities to enhance young people’s autonomy.

While adolescent friendly services, with education provided in adolescents’ own languages may be ideal, given the size of the Lao health budget, the multiple needs of the health system, and the resources and logistical challenges of providing standalone services for adolescents, in resource-limited settings, with small, geographically dispersed populations, such as in this study, it may be more appropriate to ensure that staff are trained to work with adolescents of diverse ethno-linguistic backgrounds, and are able to uphold the principles of confidentiality and privacy to make existing services more acceptable to adolescents. Specific strategies should be developed at the policy, service provider and community level and should include adolescents, families, and community leaders. Examples of some of the strategies that could be implemented ae contained in Table 2. Strategies should be designed with adolescents and take into account the normative contexts surrounding teen pregnancy, in both mainstream and minority cultures. As social norms are a group-level phenomenon [43], strategies need to target different levels of the systems as illustrated in Table 2.

**Table 2:** Examples of strategies that could reduce the incidence of teenage pregnancy

Policy level	
Ensure adolescent rights to sexual and reproductive health and access to information and services are protected in legislation and policy.	
Reduce direct and indirect costs for adolescents to in accessing sexual and reproductive health services, including access to contraceptives.	
Develop strategies to recruit young people, especially from ethnic backgrounds into sexual and reproductive health services.	
Service provider level	
Develop the cultural competence of healthcare staff and knowledge of adolescent needs with regards to sexual health.	
Co-design sexual and reproductive health services with adolescents.	
Develop confidence in healthcare staff to offer counselling and have confidential, non-judgmental discussions around sexual health with adolescents.	
Individual and community level	
Provide age-appropriate, gender-specific educational interventions, using traditional means of learning, for example, storytelling, providing opportunities for discussion and reflection.	
Integrate life-skills training for adolescent with regard to sexual and reproductive health into formal and non-formal education for adolescents to build confidence and negotiating skills.	
Engage community leaders and raise awareness with the community of the benefits of delaying pregnancy, staying at school and establishing strategies to help adolescents who are struggling to remain in school.	
Support parents to discuss relationships and sexual health with adolescents.	
Improve access to contraceptives at the community level.	

### Limitations

The triangulation of multiple viewpoints is a strength of this study, although care must be taken to separate the opinions of each type of key informant. Although this research provides data for understanding the experiences of teenage pregnancy and barriers in accessing health services by teen mothers, it is a qualitative study with potential biases resulting from selection issues, so its results are not representative and, therefore, not generalizable for all unmarried adolescent girls and teenage mothers in Lao PDR. Further, qualitative data could be subject to multiple interpretations and this study was undertaken by researchers who are Lao, but not from the adolescents’ culture, although they do have a long-standing research connections within the two provinces and selected districts. The use of local interpreters might have biased the information and disturbed the natural flow of the FGDs, however, we tried to minimize this by using an experienced female moderator and a female translator. Interviews were translated and it is possible that meaning could have been lost or distorted in the interpretation process.

## Conclusion

Despite a recognition globally that ensuring adolescents have accessible, affordable and appropriate access to SRH services, in many setting access remains poor [16, 17, 23]. Context-specific research and policy focus is required to identify opportunities for filling this critical gap in service provision [24, 25]. Policy-makers and healthcare providers also need to recognise that adolescents are sexual beings, and that we need to give adolescents the knowledge, tools and support to be able to keep themselves sexually safe and, ideally, delay pregnancy at least until after age 18. At the same time, it is important that interventions remain culturally respectful, while embracing contemporary aspirations and allow non-Lao-Tai ethnic groups to continue to live and flourish in the Lao PDR. Finally, while there is some descriptive research on outcomes for non-Lao-Tai adolescents, in Lao-Tai populations more evidence is needed for effective interventions that promote health and social justice.

## Abbreviations

ANC: Antenatal Care
FGD: Focus Group Discussion
HCP: Health Care provider
HIV: Human Immunodeficiency Virus
IDI: In-Depth Interview
Lao PDR: Lao People’s Democratic Republic
LWU: Lao Women’s Union
LYU: Lao Youth Union
MDG: Millennium Development Goal
MMR: Maternal Mortality Rate
PNC: Postnatal Care
SRH: Sexual and Reproductive Health
STI: Sexually Transmitted Infection
TBA: Traditional Birth Attendant
VHV: Village Health Volunteer
